# Early-Life Demographic Factors Shape Gut Microbiome Patterns Associated with Rotavirus Gastroenteritis Severity

**DOI:** 10.3390/v17121542

**Published:** 2025-11-26

**Authors:** Eman R. Abdelbary, Mohammed Ramadan, Ibrahim A. Amin, Fatma S. Abd-Elsamea, Ashraf Mohamed Elsaghier, Eman Ahmed Abd-Alrahman, Hani A. Ozbak, Hassan A. Hemeg, Yahya A. Almutawif, Shadi A. Zakai, Ali A. Abdelrahman, Mohammed Salah

**Affiliations:** 1 Department of Microbiology and Immunology, Faculty of Pharmacy, Al-Azhar University, Assiut 71526, Egypt; 2 Department of Medical Microbiology and Immunology, Faculty of Medicine, Assiut University, Assiut 71515, Egypt; 3 Department of Pediatrics, Gastroenterology and Hepatology Unit, Assiut University Children Hospital, Faculty of Medicine, Assiut University, Assiut 71515, Egypt; 4 Medical Microbiology and Immunology, Faculty of Medicine for Girls, Al-Azhar University, Assiut 71526, Egypt; 5 Department of Clinical Laboratory Sciences, College of Applied Medical Sciences, Taibah University, Madinah 42353, Saudi Arabia; 6 Department of Clinical Microbiology and Immunology, Faculty of Medicine, King Abdulaziz University, Jeddah 21589, Saudi Arabia; 7 Department of Microbiology and Immunology, Faculty of Pharmacy, Suez Canal University, Ismailia 41522, Egypt; 8 Department of Microbiology and Immunology, Faculty of Pharmacy, Port-Said University, Port-Said 42526, Egypt; mohamed.salah@pharm.psu.edu.eg

**Keywords:** gut microbiome, rotavirus, dysbiosis, microbial biomarkers, demographic factors, breastfeeding, cesarean delivery

## Abstract

Background: Rotavirus gastroenteritis (RVGE) remains a leading cause of severe infant diarrhea worldwide, with growing evidence supporting the role of the gut microbiome in modulating the disease. However, the interplay between early-life demographic factors, the gut microbiome, and their combined impact on RVGE clinical severity remains inadequately characterized, particularly in specific geographic populations. Aim: We aimed to investigate how demographic determinants shape gut microbiome composition and function in RVGE and how these features relate to clinical severity. Methods: In our comprehensive case–control study of 165 infants (120 RVGE cases and 45 healthy controls, aged 0–12 months), we utilized 16S rRNA sequencing combined with advanced statistical modeling and machine learning to investigate how demographic factors influence microbiome composition and clinical outcomes. Results: RVGE cases exhibited significantly reduced bacterial diversity (Kruskal–Wallis, Static = 14.85, *p* < 0.001) and distinct patterns, with community structure most strongly associated with dehydration severity (PERMANOVA; R^2^ = 0.15, *p* < 0.001). Substantial taxonomic alterations were identified characterized by depletion of beneficial commensals including *Akkermansia* (LDA score = 3.8, *p* < 0.001), *Faecalibacterium* (Random Forest AUC = 0.82, *p* < 0.001), and *Bifidobacterium* (r = −0.42 with breastfeeding, *p* < 0.001), alongside enrichment of inflammation-associated taxa such as *Escherichia*-Shigella (WBC; r = 0.49, *p* < 0.001, and CRP; r = 0.56, *p* < 0.001), Streptococcus (LDA score = 4.2, *p* < 0.001), and *Staphylococcus*. Proteobacteria was the top potential biomarker of severe outcomes (Random Forest AUC = 0.85), with abundance positively correlated with systemic inflammation (CRP: r = 0.51, *p* = 0.003). Functional predictions revealed increased lipopolysaccharide biosynthesis (ko00540) and reduced butanoate metabolism (ko00650, *p* < 0.001) in severe disease. Importantly, demographic factors significantly modulated clinical outcomes: cesarean-delivered, formula-fed infants presented the most dysbiotic profiles and experienced 3.2-fold longer hospitalization (95% CI: 1.8–5.6, *p* < 0.001) than vaginally delivered, breastfed infants did. Conclusions: Collectively, these findings demonstrate that early-life demographic factors potentially shape the gut microbiome composition and function, may influence RVGE severity and recovery trajectories, thus providing candidate biomarkers for risk stratification and identifying targets for microbiota-based interventions.

## 1. Introduction

Rotavirus remains the leading global cause of severe dehydrating gastroenteritis in children under five years of age, accounting for approximately 128,000 annual deaths, predominantly in low- and middle-income countries [1,2]. Despite the introduction of vaccines, rotavirus continues to cause substantial morbidity, with emerging evidence suggesting that host susceptibility and disease severity may be modulated by the gut microbiome [3]. The gut microbiota plays a pivotal role in immune system development, pathogen resistance, and metabolic homeostasis early in life, with perturbations linked to both acute infectious outcomes and long-term health consequences [4,5]. Understanding how rotavirus infection interacts with the developing gut microbiome is critical for improving therapeutic strategies and identifying at-risk populations.

The infant gut microbiome matures rapidly in the first two years and is shaped by delivery mode, feeding, antibiotic exposure, and geography [6,7]. Specific practices, such as exclusive breastfeeding, have been shown to foster microbial communities that enhance gut barrier integrity and immune tolerance [8,9]. Conversely, cesarean section delivery and formula feeding are associated with delayed microbial diversification and altered colonization patterns [10,11]. These early-life microbial disturbances have been linked to an increased susceptibility to enteric infections, including rotavirus, although the precise mechanisms underlying this association remain incompletely understood [12].

Rotavirus infection itself induces significant alterations in the gut microbial composition, characterized by decreased diversity and enrichment of proinflammatory taxa [13]. Studies in murine models have demonstrated that rotavirus disrupts the intestinal epithelial barrier, promoting bacterial translocation and systemic inflammation [14]. In humans, rotavirus-positive infants exhibit specific alterations in microbial composition that correlate with prolonged diarrhea and severe dehydration [15]. These findings suggest that rotavirus may exploit or exacerbate preexisting dysbiosis, creating a vicious cycle of inflammation and impaired microbial recovery [16].

The bidirectional relationship between rotavirus and the gut microbiome is further complicated by demographic and environmental variables. Geographic location has been shown to influence microbial community structure, which may affect disease outcomes [17,18]. Urban and rural environments appear to shape distinct microbial profiles, often characterized by differences in microbial diversity, the abundance of immunomodulatory commensals, and exposure to environmental microbes, that differentially influence susceptibility to infection by priming the host immune system and altering colonization resistance against pathogens [19,20,21]. Socioeconomic factors, including access to clean water and vaccination coverage, further modulate these interactions, highlighting the need for region-specific microbiome studies in rotavirus-endemic areas [22].

Emerging evidence suggests that microbiome-targeted interventions could ameliorate rotavirus severity by restoring microbial homeostasis [23,24]. Specific microbial strains have shown efficacy in reducing diarrhea duration and viral shedding in clinical trials, likely through immune modulation and competitive exclusion of pathogens [22,25]. However, the variable success of these interventions underscores the importance of personalized approaches, accounting for the host microbiome baseline and demographic risk factors [26]. Evidence from numerous clinical trials and systematic reviews supports the use of specific probiotic strains as an adjuvant therapy for acute rotavirus gastroenteritis in children [27,28]. Large-scale meta-analyses, including a Cochrane review of 63 RCTs, conclude that probiotics can reduce the mean duration of diarrhea by approximately 25 h and the risk of prolonged episodes by 59%. The most robust evidence supports *Lacticaseibacillus rhamnosus* GG (LGG) and *Saccharomyces boulardii*, which consistently demonstrate significant reductions in diarrhea duration and severity in various clinical settings [27]. The efficacy is strain and dose-dependent, leading to recommendations from bodies like ESPGHAN for the use of these specific probiotics as a viable, evidence-based approach to hasten recovery when administered early in the illness [29].

A significant gap remains in understanding how specific early-life demographic factors interact with the gut microbiome to modulate the severity of rotavirus gastroenteritis (RVGE) across distinct populations. This study aims to address that gap by integrating detailed demographic data with microbiome analysis to identify predictive biomarkers and potential therapeutic targets. Using 16S rRNA sequencing and clinical metadata, we investigated how demographic factors influence gut microbiome composition and function in RVGE and how these microbial features correlate with clinical severity.

## 2. Materials and Methods

### 2.1. Participants and Eligibility Criteria

This case–control study was conducted between November 2022 and June 2023 at Assiut University Children’s Hospital, specifically within the Gastroenterology Unit and the Neonatal Intensive Care Unit. Infants aged 0 to 12 months who were presented with acute gastroenteritis were screened for enrollment.

Infants were eligible for inclusion if they were between 0 and 12 months of age. The case group (RVGE) consisted of infants diagnosed with acute gastroenteritis, defined as having three or more watery stools per day for less than 72 h, along with laboratory-confirmed rotavirus infection. Confirmation was achieved through a stool antigen test (Rotaclone ELISA, Meridian Bioscience; sensitivity 98.4%, specificity 99.3%) and/or quantitative RT-PCR using the CDC Rotavirus Assay targeting the VP6 gene [30]. The control group included healthy infants attending well-baby clinics (routine health check-up visits for growth and development monitoring) who had not experienced any diarrhea in the preceding month. These controls were frequency-matched to the cases by age band and were recruited from the same geographic catchment area to minimize socioeconomic and environmental confounding. Written informed consent was obtained from the parents or legal guardians of all participants for both participation and stool microbiome analysis. Crucially, no participants (cases or controls) had received rotavirus vaccination before enrollment, to eliminate potential confounding effects of vaccine-induced immune and microbiome modulation on disease presentation and severity. This is because the rotavirus vaccine was not included in the national immunization program in Egypt at the time of this study, ensuring a uniformly unvaccinated cohort.

Exclusion criteria included the use of antibiotics, probiotics, or prebiotics within 14 days prior to enrollment, assessed through a combination of parent interviews and medical record verification, when available. Infants with chronic gastrointestinal disorders such as inflammatory bowel disease, cystic fibrosis, or congenital malformations affecting digestion were excluded. Additional exclusions applied to those with severe comorbidities, including congenital heart disease, chronic kidney disease, or immunodeficiency. Infants with other enteric infections detected by stool culture or PCR, whether bacterial, viral, or parasitic, were also excluded. Premature infants (born before 37 weeks of gestation) or those with very low birth weight (less than 1500 g) were not eligible. Hospitalization within the preceding month, except for delivery, was another exclusion criterion, as was the presence of incomplete demographic or clinical data or inadequate stool samples for microbiome sequencing. Samples with fewer than 10,000 reads after quality control were excluded from the analysis (*n* = 2). No imputation was performed for missing clinical or demographic data; complete case analysis was used Length of stay (LOS) was calculated as the number of days from hospital admission to discharge. LOS data were collected from electronic medical records and verified through nursing discharge summaries. Prolonged hospitalization was defined as LOS ≥ 7 days based on clinical guidelines for pediatric gastroenteritis management and the distribution of LOS in our cohort, which identified this threshold as clinically meaningful for distinguishing extended recovery [31]. Multivariate regression analysis was performed to identify demographic and clinical predictors of extended LOS, adjusting for age, sex, residence, and disease severity.

### 2.2. Clinical Assessment

Disease severity was assessed using the Vesikari clinical severity scoring system (severe ≥ 11) by trained pediatricians affiliated with the study [32]. In addition, dehydration grade (none, moderate, severe) was recorded at presentation and used as a severity classifier in pre-specified analyses [33]. Laboratory measures included C-reactive protein (CRP), electrolytes, complete blood counts, and other routine parameters as indicated [34]. Analyses involving dehydration severity were conducted within the RVGE cohort only to avoid trivial separation from healthy controls (who, by definition, had no dehydration).

### 2.3. Sample Collection, PCR Amplification, and Sequencing of 16S rRNA

Owing to the complex nature of microbial data and variability across sequencing platforms, the sample size was guided by established recommendations and prior studies reporting Shannon diversity values between 2.1 and 3.2 in infant gut microbiomes affected by gastroenteritis. Recent simulation-based evaluations suggest that detecting medium effect sizes (Cohen’s d ≈ 0.5–0.6) in Shannon diversity or the relative abundance of dominant taxa typically requires 40–60 subjects per group to achieve 80% power at α = 0.05 [35,36,37].

Stool samples were collected within 24 h of hospitalization and control stools were collected at the clinic visit, frozen at −80 °C, and processed for DNA extraction using the Qiagen DNeasy PowerSoil Kit (Cat No: 47014; Qiagen, Hilden, Germany) within 4 weeks to minimize degradation. DNA quality and quantity were assessed using a NanoDrop spectrophotometer and Qubit fluorometer, respectively, before library preparation. The V3–V4 region of the 16S rRNA gene was amplified through the following primers with Illumina adapter overhangs (underlined): forward primer 5′ TCGTCGGCAGCGTCAGATGTGTATAAGAGACAGCCTACGGGNGGCWGCAG 3′ reverse primer 5′ GTCTCGTGGGCTCGGAGATGTGTATAAGAGACAGGACTACHVGGGTATCTAATCC 3′. Polymerase chain reaction (PCR) was performed in a final volume of 25 μL. Each reaction mixture comprised 0.8 μL of forward primer and 0.8 μL of reverse primer, both at a concentration of 10 μM (Metabion, Planegg, Germany), 3 μL of template DNA, and 12.5 μL of 1× Hot Master Mix (Genedirex PCR Supermix). The thermal cycling conditions included an initial denaturation at 94 °C for 3 min, followed by 22 cycles of denaturation at 95 °C for 30 s, annealing at 60 °C for 30 s, and extension at 72 °C for 30 s. A final extension step was carried out at 72 °C for 5 min.

The amplicon size and quality were verified through 1% agarose gel electrophoresis. Library preparation and PCR amplicon processing were performed by IGA Technology Services (Udine, Italy), and sequencing was carried out on the Illumina MiSeq platform (Illumina, San Diego, CA, USA) [38]. Negative extraction controls and PCR blanks were incorporated into every batch and sequenced concurrently with the samples, yielding no detectable reads after quality filtering. All sample handling and DNA extraction procedures were performed in compliance with institutional biosafety protocols, with stool samples treated as potential biohazards and processed using appropriate personal protective equipment within a Class II biosafety cabinet. This quality control measure ensures that the detected microbial profiles reflect genuine biological signals instead of artifacts introduced during the sample processing phase. The ethics committee approved the study protocol at the Faculty of Pharmacy, Al-Azhar University, Assiut, Egypt (ZA-AS/PH/12/C/2022), adhering to the Declaration of Helsinki guidelines [39].

### 2.4. Bioinformatic Processing and Taxonomic Classification

The bioinformatics pipeline for processing 16S rRNA gene sequencing data was designed to maximize accuracy while minimizing technical biases. Raw paired-end FASTQ files were first subjected to quality control through FastQC (v0.11.9) to assess sequence quality distributions. Sequence processing used the QIIME2 pipeline (2025.4) with the DADA2 plugin [40]. Quality filtering was applied to discard reads with expected errors > 2 in either the forward or reverse read, followed by truncation of forward reads at 270 bp and reverse reads at 210 bp based on Phred quality score degradation profiles (Phred quality ≥ 25) [41].

The denoised and merged sequences were clustered into amplicon sequence variants (ASVs) through DADA2. Chimeric sequences were detected and removed through the consensus method. Taxonomic classification was performed by applying a pretrained naive Bayesian classifier (v2.20.0) against the SILVA 138.1 reference database [42,43]. Techniques from machine learning have been utilized and validated for research on microbiomes to identify microbial signatures that differentiate between comparable groups [44]. Random-forest classifiers (ntree = 500; max_depth = 5) were evaluated with nested cross-validation (5 × 10 folds) and 1000 permutations. To identify significant bacterial indicators, we also used the random forest classifier through MicrobiomeAnalyst [45,46]. The validation of the model included (1) nested cross-validation with 5 outer folds and 10 inner folds to assess the AUC, precision-recall, and calibration; (2) testing independent holdout samples, which represented 30% of the data; and (3) conducting 1000 permutation runs to verify robustness. To ensure comprehensive performance evaluation, we implemented rigorous validation metrics including bootstrap-derived confidence intervals for all Area Under the Curve (AUC) values (1000 iterations with bias-corrected accelerated intervals). Feature importance was quantified using Mean Decrease Gini impurity scores, with top discriminative taxa ranked by their contribution to classification accuracy. Additional performance metrics including precision, recall, F1-scores, and multiclass accuracy were calculated with 95% confidence intervals. Model robustness was further validated through permutation testing (1000 iterations) to establish statistical significance beyond chance classification. The functional profile of the gut microbiome was inferred from 16S rRNA data (V3-V4 region) through Tax4Fun [47], with the Kyoto Encyclopedia of Genes and Genomes (KEGG) Orthologs (KOs) database informing the analysis [48]. Functional pathway abundances predicted by Tax4Fun were normalized using total sum scaling (TSS) before downstream statistical analysis.

### 2.5. Statistical Analysis

Comprehensive statistical analyses were conducted to examine relationships between microbial community features and demographic/clinical variables. Age distribution was compared using Fisher’s exact test on collapsed categories (0–3, 4–6, 7–9, and 10–12 months) due to low expected frequencies in monthly strata. Alpha diversity metrics (Shannon index, observed ASVs, and Chao1) and beta diversity were calculated using the phyloseq package (v1.36.0) at a standardized sequencing depth of 26,908 reads per sample to preserve diversity metrics (Supplementary Figure S1) [49,50]. In addition to standard alpha diversity metrics (Shannon, Chao1, observed ASVs), we calculated the Berger–Parker index to assess community dominance, defined as the proportional abundance of the most dominant Amplicon Sequence Variant (ASV) in a sample. Group-wise comparisons of the Berger–Parker index were conducted using nonparametric tests (Wilcoxon rank-sum test for two groups, Kruskal–Wallis test for multiple groups) to evaluate differences in dominance patterns across clinical and demographic categories [51]. Beta diversity was assessed through principal coordinate analysis (PCoA) of Bray–Curtis dissimilarity matrices, and statistical significance of group separations was tested using permutational multivariate analysis of variance (PERMANOVA) with 999 permutations [52]. A multi-faceted statistical approach was employed to address distinct biological questions and leverage the strengths of different tools, ensuring robust and comprehensive analysis. Each tool was applied consistently with its data type and analytical goal: DESeq2 was used for differential abundance testing of raw counts between predefined groups; LEfSe identified biomarkers distinguishing multiple (>2) categorical classes; MaAsLin2 detected multivariate associations while controlling for covariates (e.g., age, sex); Spearman correlation assessed monotonic relationships between microbial relative abundance and continuous clinical variables; and Random Forest machine learning evaluated predictive power of the entire microbial community and ranked feature importance. This integrated approach enabled cross-validation of findings across complementary methodologies. Differential abundance analysis was performed on nonrarefied counts at multiple taxonomic levels through DESeq2 (v1.32.0), which employs a negative binomial generalized linear model with Wald tests for significance [53]. Additionally, linear discriminant analysis (LDA) effective size (LEfSe) was applied to infer potential biomarkers (taxon or metabolic pathways) related to health states and demographic and clinical variables (LDA score ≥ 2.0, *p* < 0.05) [54]. Microbial association analysis was conducted through Maaslin2 (Multivariate Association with Linear Models), which employs a linear modeling approach to identify taxa significantly associated with clinical groups [55]. The genus-level ASV table served as the input data, while the metadata encompassed disease state, clinical variables, demographics, age, and sex. These factors were included as fixed effects in the model to account for demographic confounding. To stabilize variance and mitigate compositional bias, total sum scaling (TSS) normalization and log transformation were applied. To define the strength of associations, Spearman’s correlation coefficients (*r*) were categorized as follows: weak (0.20–0.39), moderate (0.40–0.59), and strong (≥0.60). For all the statistical tests, a two-tailed *p* value of less than 0.05 was considered statistically significant. *p* values were further denoted as follows: * *p* < 0.05, ** *p* < 0.01, and *** *p* < 0.001.

The Firmicutes-to-Bacteroidetes (F/B) ratio was log-transformed to approximate normality and analyzed in relation to demographic factors through multiple linear regression models adjusted for age, sex, and feeding mode. Continuous variables were standardized (through a z-score transformation) to facilitate comparison of effect sizes. Pairwise comparisons were statistically estimated through an unpaired Wilcoxon rank sum test, while multiple-testing-group comparisons were performed through the Kruskal–Wallis rank sum test, and correction of *p* values was applied through the Benjamini–Hochberg false discovery rate (FDR) procedure [56]. *p*-values from differential abundance testing (DESeq2, MaAsLin2), functional pathway analyses, and genus-level Spearman correlations were adjusted for multiple comparisons using the Benjamini–Hochberg FDR procedure. An FDR-adjusted *p*-value (q-value) of < 0.05 was considered statistically significant. *p* values resulting from statistical tests that were below the threshold of 0.001 are reported as *p* < 0.001. All analyses were conducted in R (v4.2.2) [57]. In summary, key associations were tested using multivariate models adjusted for covariates where specified, and statistical significance was interpreted in the context of FDR correction for multiple comparisons where applicable.

## 3. Results

### 3.1. Demographic and Clinical Characteristics of the Participants

The study cohort comprised 165 infants, including 120 with confirmed rotavirus gastroenteritis (RVGE) and 45 healthy controls. The patients’ demographic and clinical characteristics are summarized in Table 1 (Supplementary Table S1). The groups were comparable in terms of sex distribution (37.5% male in RVGE vs. 46.7% in controls; χ^2^ = 1.12, *p* = 0.29) and residential setting (40.0% rural in RVGE vs. 48.9% in controls; χ^2^ = 1.06, *p* = 0.30). Age distribution did not differ significantly between groups (Fisher’s exact test, *p* = 0.065) (Supplementary Table S1).

However, feeding practices differed markedly, with the RVGE group having a significantly greater proportion of formula-fed infants (46.7% vs. 20.0% in controls; χ^2^ = 9.87, *p* = 0.002) and a lower proportion of exclusively breastfed infants (48.3% vs. 68.9%). The mixed feeding category comprised 6 infants (5.0%) in the RVGE group compared to 5 (11.1%) in controls. The mode of delivery did not differ significantly between the groups (70.8% cesarean in the RVGE group vs. 75.6% in the control group; χ^2^ = 0.31, *p* = 0.58).

As expected, clinical parameters reflecting infection and inflammation were significantly altered in the RVGE cohort. RVGE infants presented elevated white blood cell (WBC) counts (11.2 ± 3.1 vs. 6.1 ± 2.0 × 10^3^/µL; *p* < 0.001), lower hemoglobin levels (11.0 ± 1.3 vs. 12.3 ± 0.8 g/dL; *p* < 0.001), and higher C-reactive protein (CRP) concentrations (median [IQR]: 1.6 [0.9–2.2] vs. 0.5 [0.3–0.7] mg/L; *p* < 0.001). Dehydration was present in 70.0% of cases (63.3% moderate, 6.7% severe; χ^2^ = 72.0, *p* < 0.001).

Hospitalization outcomes revealed significant variations in length of stay (LOS) across demographic subgroups (Supplementary Table S2). Cesarean-delivered, formula-fed infants (32.50%) experienced substantially longer hospitalization (7.9 ± 1.2 days) compared to vaginally delivered, breastfed infants (13.33%; 2.4 ± 1.1 days), representing a 3.3-fold increase (*p* < 0.001). Mixed feeding practices showed intermediate outcomes, with vaginal + mixed infants (1.67%) requiring 3.8 ± 1.0 days (1.6-fold increase, *p* = 0.08) and cesarean + mixed infants (3.33%) requiring 5.2 ± 1.1 days (2.2-fold increase, *p* = 0.01). LOS showed a strong correlation with clinical severity, with severely dehydrated infants (6.67%) requiring 6.8 ± 1.7 days versus 2.0 ± 0.5 days for non-dehydrated cases (30.00%; *p* < 0.001).

### 3.2. Bacterial Diversity Analysis

**Figure 1:**
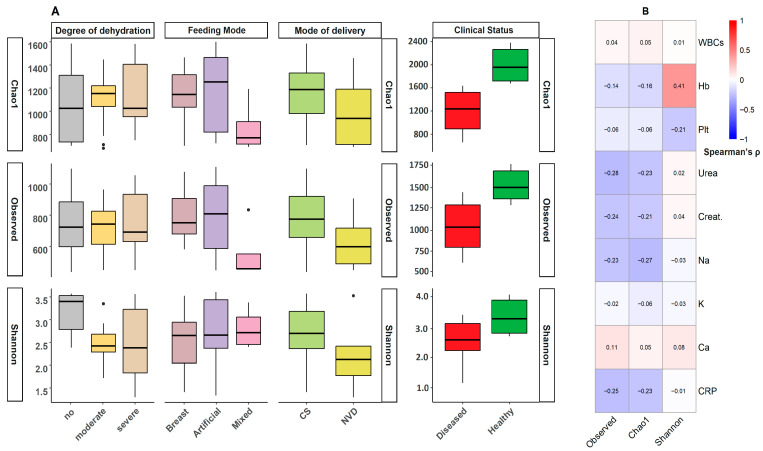
Bacterial diversity analysis and Correlation patterns between clinical parameters and gut microbiome diversity. The figure presents a comprehensive analysis of the relationships between host parameters and the alpha diversity of the gut microbiome. (**A**) Boxplots of three alpha diversity metrics (Shannon, Chao1, and observed species) stratified by four clinical/demographic parameters (Disease state, feeding practices, delivery mode, and dehydration status). (**B**) Clustered heatmap of Spearman correlation coefficients between clinical parameters (including hematological and biochemical measures) and alpha diversity indices. The heatmap employs a red white–blue color gradient (red = 1, white = 0, blue = −1). All correlation values are displayed numerically (*p* < 0.05). Abbreviations: Plt, Platelets; Ca, Calcium; Na, Sodium; CRP, C-reactive protein; WBC, White Blood Cell count; Hb, Hemoglobin; Creat., Creatinine.

Sequencing of 16S rRNA amplicons generated 6,435,520 high-quality reads from 165 samples (mean ± SD: 37,856 ± 10,737 reads per sample). Post-DADA2 processing, chimera removal accounted for <12.73% of sequences. Negative controls yielded no detectable reads. Rarefaction curves in Supplementary Figure S1 demonstrate adequate sequencing depth. Alpha diversity was significantly lower in the RVGE cohort than in the healthy control cohort (Kruskal–Wallis, Static = 14.85, *p* < 0.001; Figure 1A).

Within the RVGE cohort, alpha diversity was significantly associated with several host factors. Feeding practices were a major determinant, with a significant difference in Shannon diversity across the three groups (Kruskal–Wallis test, Static = 21.73, *p* = 0.015; Figure 1B). Breastfed infants had significantly greater diversity than formula-fed infants (Dunn’s test, *p* = 0.018). Similarly, the mode of delivery influenced diversity, with vaginally delivered infants exhibiting higher values than those delivered by cesarean section (Wilcoxon rank-sum test, *p* = 0.022). A significant negative correlation was also observed between diversity and the duration of formula feeding (*r* = −0.38, *p* = 0.004).

Clinical disease severity was strongly associated with a loss of diversity. Shannon diversity was significantly negatively correlated with both white blood cell count (*r* = −0.35, *p* = 0.008) and CRP level (*r* = −0.42, *p* < 0.001). Most notably, diversity decreased significantly with increasing dehydration grade (Kruskal–Wallis test, Static = 25.8, *p* < 0.001), with severely dehydrated infants exhibiting the lowest microbial diversity. Age was positively correlated with diversity (*r* = 0.39, *p* = 0.002), whereas rural residence had a modest negative association (*r* = −0.27, *p* = 0.032). No significant correlations were found with hemoglobin or platelet counts.

Analysis of microbial community dominance using the Berger–Parker Index revealed distinct patterns across demographic and clinical variables (Supplementary Table S3). RVGE patients exhibited substantially higher dominance (mean = 0.42) than healthy controls (mean = 0.27), indicating a collapse in community evenness characterized by pathogen overgrowth during infection. Among feeding practices, breastfed infants showed the highest dominance (0.47), potentially reflecting specialized colonization by milk-adapted taxa, while mixed-fed infants displayed the most even distributions (0.29). In contrast, geographic residence showed no influence on community dominance, with nearly identical indices between rural (0.42) and urban (0.39) infants.

**Figure 2:**
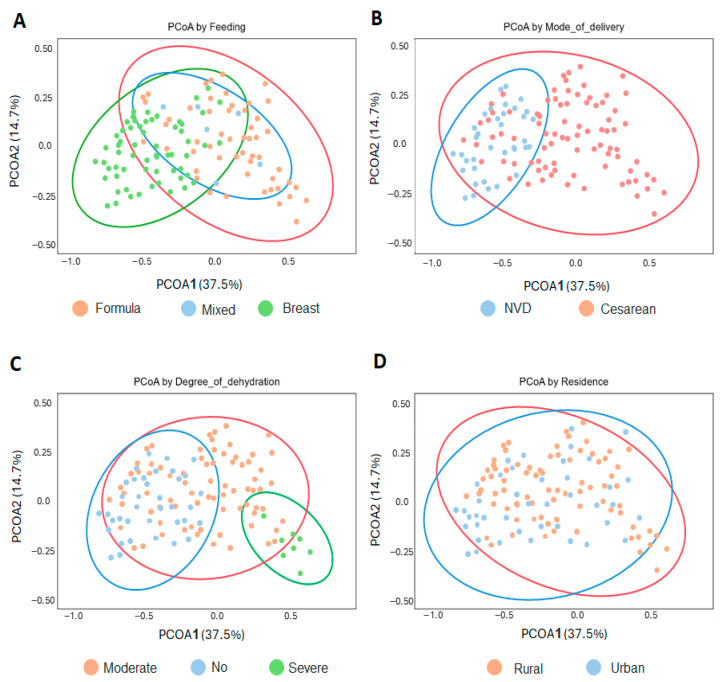
Principal coordinate analysis reveals associations between the infant gut microbiota and demographic and clinical variables. Principal coordinate analysis (PCoA) based on Bray–Curtis dissimilarity metrics illustrating gut microbiota β diversity stratified by (**A**) feeding mode, (**B**) mode of delivery, (**C**) degree of dehydration, and (**D**) place of residence. Each point represents an individual infant, with colors indicating the category of each variable. The ellipses denote 95% confidence intervals for the group centroids. Variations in microbial community composition were visualized along the first two principal coordinates (PCoA1 and PCoA2), which explained 37.5% and 14.7% of the total variance, respectively (PERMANOVA; R^2^ = 0.15, *p* < 0.001).

Notably, the gut microbiome composition of RVGE infants was significantly associated with key demographic and clinical factors (PERMANOVA: FDR-adjusted *p* < 0.05) (Figure 2). The degree of dehydration was the strongest determinant of microbial structure (R^2^ = 0.15, *p* < 0.001), followed by feeding mode (R^2^ = 0.12, *p* < 0.001), delivery mode (R^2^ = 0.08, *p* = 0.02), and residential environment (R^2^ = 0.06, *p* = 0.04).

### 3.3. Taxonomic Profiling of RVGE Microbiomes: Phylum-Level Associations with Demographic and Clinical Variables

Analysis of the 1247 bacterial ASVs revealed a taxonomic structure comprising 17 phyla, 52 classes, 127 orders, 231 families, and 419 genera.

**Figure 3:**
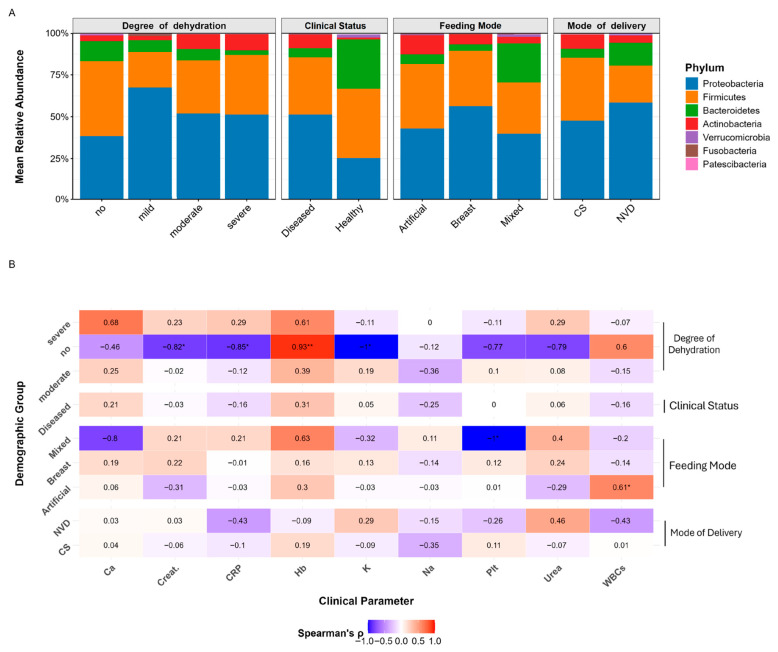
Microbiome composition and Firmicutes-to-Bacteroidetes ratio associated with clinical and demographic variables in the study cohort. Analysis of the gut microbiota composition and its clinical associations, integrating taxonomic profiling with functional dysbiosis metrics. (**A**) Bar plots displaying the mean relative abundance of the most dominant bacterial phyla across demographic groups. (**B**) Heatmap showing Spearman correlations between the Firmicutes-to-Bacteroidetes ratio (F/B ratio) and clinical parameters. Correlation coefficients (*r*) are displayed with significance indicators (* *p* < 0.05, ** *p* < 0.01). Red/blue gradients denote positive/negative associations, respectively. Abbreviations: Plt, Platelets; Ca, Calcium; Na, Sodium; CRP, C-reactive protein; WBC, White Blood Cell count; Hb, Hemoglobin; Creat., Creatinine.

Phylum-level abundance was strongly associated with clinical and demographic factors (Figure 3A). Firmicutes was the dominant phylum (mean abundance: 38.6%). Its relative abundance increased significantly with increasing age (*r* = 0.42, *p* = 0.008) and was greater in breastfed infants than in formula-fed infants (Kruskal–Wallis test followed by Dunn’s post hoc test, *p* = 0.045).

Proteobacteria, the second most abundant phylum (32.1%), was strongly associated with disease severity. It was significantly enriched in infants with severe dehydration (Kruskal–Wallis test, *p* = 0.012; post hoc severe vs. no dehydration: *p* = 0.009), and its abundance correlated positively with systemic inflammation (CRP: *r* = 0.51, *p* = 0.003). This clinical relevance was further underscored by machine learning, which identified Proteobacteria as the top classifier for severe outcomes (AUC = 0.85).

In contrast, Actinobacteria (10.2%) and Bacteroidetes (7.8%) exhibited protective associations. Actinobacteria was enriched in breastfed infants (Kruskal–Wallis, *p* = 0.008; post hoc *p* = 0.006) and was negatively correlated with dehydration severity (*r* = −0.35, *p* = 0.018). The abundance of Bacteroidetes was significantly lower in patients with severe dehydration (Kruskal–Wallis, *p* = 0.023; post hoc *p* = 0.018) and was negatively correlated with age (*r* = −0.38, *p* = 0.012). Compositional plots visually confirmed the greater contribution of these phyla to the microbiomes of healthy and breastfed infants.

The Firmicutes-to-Bacteroidetes (F/B) ratio, a common index of dysbiosis, varied significantly across groups (Figure 3B). The ratio was elevated in severely dehydrated infants (Kruskal–Wallis, *p* = 0.032), which is consistent with the observed enrichment of Firmicutes and depletion of Bacteroidetes. Formula-fed infants had a significantly higher median F/B ratio than their breastfed counterparts did (2.01 vs. 1.24; Wilcoxon rank-sum test, *p* = 0.018). Older infants (>6 months) also presented higher ratios than younger infants did (median: 2.34 vs. 1.15; Wilcoxon test, *p* = 0.047), whereas a nonsignificant trend toward higher ratios was observed in rural versus urban infants (1.71 vs. 1.29; *p* = 0.12).

### 3.4. Microbial Signatures: Genus-Level Stratification Reveals Demographic Patterns in RVGE

**Figure 4:**
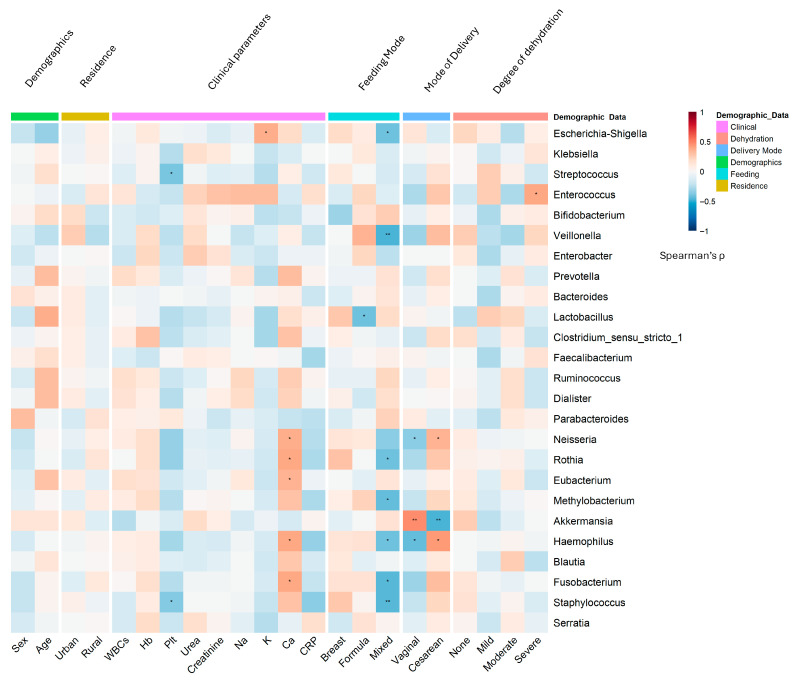
Gut microbiome structure at genus level in rotavirus gastroenteritis (RVGE): Associations with demographic and clinical parameters. Heatmap visualization of Spearman correlation coefficients between the relative abundance of the top 25 bacterial genera (Mean relative abundance ≥ 0.02) (*y*-axis) and demographic/clinical variables (*x*-axis). The variables are grouped by type (feeding, delivery mode, demographics, clinical, dehydration). The color intensity represents the correlation strength (blue: negative; red: positive; scale: −1 to +1). Asterisks denote statistical significance (* *p* < 0.05, ** *p* < 0.01) based on two-tailed tests. Abbreviations: Plt, Platelets; Ca, Calcium; Na, Sodium; CRP, C-reactive protein; WBC, White Blood Cell count; Hb, Hemoglobin; Creat., Creatinine.

The gut microbiota of infants with RVGE was characterized by significant genus-level dysbiosis, the patterns of which were strongly influenced by host demographics (Figure 4).

A consistent depletion of beneficial, anti-inflammatory genera was observed. This included a significant reduction in *Akkermansia* (LDA score = 3.8, *p* < 0.001), which was negatively correlated with CRP levels (*r* = −0.45, *p* < 0.001). The abundances of the butyrate producers *Roseburia* (DESeq2 log2FC = −2.1, *p* = 0.003) and *Eubacterium* (MaAsLin2 coefficient = −0.67, *p* = 0.0087) were also markedly reduced.

Conversely, opportunistic pathogens were enriched. *Streptococcus* was identified as a potential dysbiosis biomarker (LDA score = 4.2, *p* < 0.001) and was strongly associated with formula feeding and elevated CRP. *Staphylococcus* (DESeq2 log2FC = 1.9, *p* = 0.004) and *Enterococcus* were both linked to cesarean delivery (*r* = 0.39, *p* = 0.002 and *r* = 0.41, *p* < 0.001, respectively).

### 3.5. Influence of Demographic Factors on Microbiome Structure

**Figure 5:**
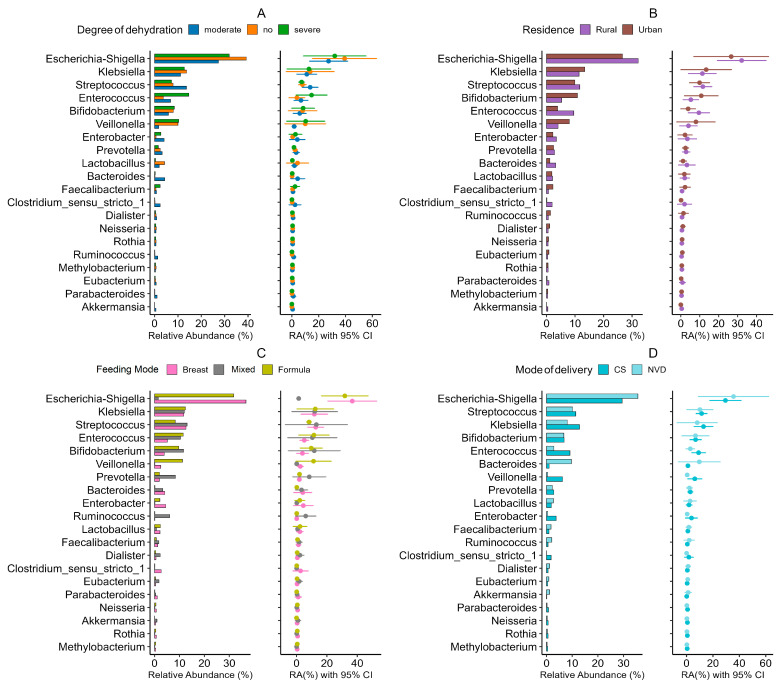
Gut microbial composition by demographic and clinical factors in the RVGE cohort. A multipanel figure presents the relative abundance patterns of the most variable bacterial genera across four demographic and clinical variables: degree of dehydration (**A**), residence type (**B**), feeding mode (**C**), and mode of delivery (**D**). Horizontal bar plots show the mean relative abundance (%) of each genus, with dot plots displaying the mean abundance and 95% confidence intervals for each genus across the respective groups. Abbreviations: Plt, Platelets; Ca, Calcium; Na, Sodium; CRP, C-reactive protein; WBC, White Blood Cell count; Hb, Hemoglobin; Creat., Creatinine; CS, Cesarean; NVD, normal vaginal delivery.

- The random forest classifier demonstrated strong performance in discriminating infant feeding modes based on gut microbiome composition (Figure 5). Cross-validation revealed excellent discriminative ability for breastfed infants (AUC = 0.85, 95% CI: 0.79–0.91), very good discrimination for formula-fed infants (AUC = 0.82, 95% CI: 0.76–0.88), and good discrimination for mixed-fed infants (AUC = 0.78, 95% CI: 0.71–0.85). Overall multiclass classification achieved an AUC of 0.81 (95% CI: 0.75–0.87), indicating robust model performance (Supplementary Figure S2, Supplementary Table S4).
- Age and sex: The inflammatory genus *Escherichia-Shigella* was significantly enriched in male infants (*r* = 0.32, *p* = 0.003; DESeq2 log2FC = 2.3, *p* = 0.0026) and younger infants (<6 months; *r* = −0.41, *p* < 0.001), who also presented relatively high levels of *Clostridium sensu stricto 1* (*r* = −0.35, *p* = 0.003) (Figure 5). In contrast, female infants and older infants presented greater abundances of the beneficial genera *Bifidobacterium* (*r* = −0.35, *p* < 0.001) and *Bacteroides*, respectively.
- Residence: Rural infants presented relatively high abundances of *Clostridium sensu stricto 1* (LDA score = 3.5, *p* = 0.002) and *Klebsiella* (*r* = 0.38, *p* < 0.001) (Figure 5B).
- Delivery mode: Cesarean delivery was associated with an increased abundance of pathobionts such as *Klebsiella* (*r* = 0.45, *p* < 0.001) and *Enterococcus,* and a reduction in beneficial taxa such as *Prevotella* (MaAsLin2 coefficient = −0.89, *p* = 0.008) (Figure 5D). Vaginal delivery was associated with increased levels of *Bifidobacterium* (Supplementary Figure S3).
- Feeding Practices: Formula feeding was a major driver of dysbiosis, showing strong positive associations with *Streptococcus* (*r* = 0.47, *p* < 0.001) and *Staphylococcus* (*r* = 0.39, *p* = 0.002). Exclusive breastfeeding promoted a healthier microbiota enriched with *Bifidobacterium* (*r* = −0.42, *p* < 0.001) and *Lactobacillus* (*r* = −0.36, *p* = 0.004). Based on the AUC analysis, the *Rikenellaceae RC9 gut group* emerged as a notable discriminator for both the formula feeding (AUC = 0.758) and breastfeeding (AUC = 0.745) groups. *Dickeya* also exhibited strong performance for the formula-fed group (AUC = 0.682), whereas *Fastidiosipila* was significant for the breastfed group (AUC = 0.701) (Figure 5C; Supplementary Figure S4).

### 3.6. Synergistic Associations and Clinical Correlations with Gut Microbiomes

The combination of cesarean delivery and formula feeding had synergistic associations, amplifying dysbiosis to produce the most unfavorable microbial profile. This was characterized by a non-linear potentiation, resulting in the highest abundance of *Staphylococcus* (r = 0.42, *p* < 0.001) and the most severe depletion of *Bifidobacterium* (r = −0.51, *p* < 0.001) and *Akkermansia* (r = −0.64, *p* < 0.001). The magnitude of these shifts in the combined group substantially exceeded the individual effects of either factor alone, indicating a convergent biological impact on microbial colonization.

Strong genus-clinical correlations were evident across the cohort: *Escherichia-Shigella* abundance was positively correlated with WBC (r = 0.49, *p* < 0.001) and CRP (r = 0.56, *p* < 0.001), whereas *Bifidobacterium* and *Faecalibacterium* abundance was negatively correlated with inflammation. The presence of *Klebsiella* was strongly linked to severe dehydration (r = 0.52, *p* < 0.001).

### 3.7. Metabolic Pathway Alterations and Functional Profiles

**Figure 6:**
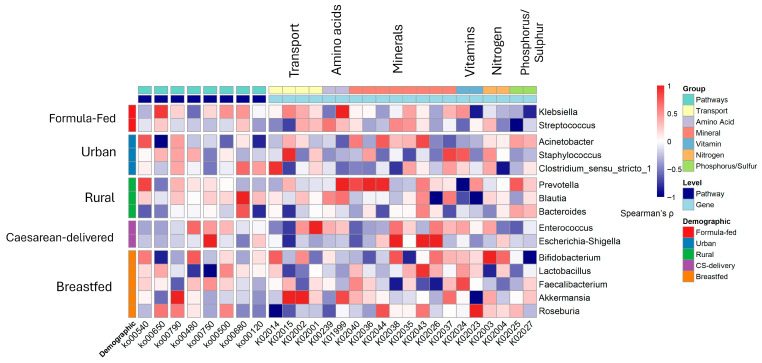
Functional correlation heatmap of microbial genera and KOs grouped by demographic variables and metabolic pathways. Heatmap illustrates the correlation patterns between significant KOs (*x*-axis) and associated microbial genera (*y*-axis), organized by demographic associations and metabolic pathways. Genera are grouped according to their predominant demographic variables (formula-fed, urban, rural, CS-delivered, and breastfed), whereas KOs are categorized by their metabolic functions. The color gradient represents correlation coefficients ranging from −1 (navy blue) to +1 (red), with white indicating a neutral correlation. The annotation bars provide immediate visual reference to the grouping structures, with genera colored by demographic association and KOs colored by metabolic pathway. LEfSe identified biomarkers distinguishing multiple categorical classes (LDA score ≥ 2.0, *p* < 0.05).

Predicted metagenomic analysis revealed profound restructuring of microbial metabolic potential in RVGE, characterized by a shift away from homeostasis and toward inflammation, with demographic factors strongly influencing these functional profiles (Figure 6, Supplementary Table S5). A core finding was the marked predicted enrichment of proinflammatory pathways, including lipopolysaccharide (LPS) biosynthesis (ko00540), which was significantly enriched in the RVGE cohort (LDA score = 4.1, *p* < 0.001) and among formula-fed infants (LDA score = 3.8, *p* = 0.002). Concurrently, pathways for xenobiotic degradation (ko00480) were elevated in cesarean-delivered infants (LDA score = 3.5, *p* = 0.007).

In contrast, protective metabolic pathways were significantly depleted. Butanoate metabolism (ko00650) was reduced in severely dehydrated patients (LDA score = 4.5, *p* < 0.001), correlating with taxonomic predicted depletion of key butyrate producers like *Faecalibacterium* (r = 0.52, *p* = 0.003). Similarly, folate biosynthesis pathways (ko00790) were underrepresented in RVGE infants (LDA score = 3.2, *p* = 0.009), aligning with lower abundances of *Bifidobacterium*.

Demographic factors further shaped the functional landscape. Breastfed infants showed enrichment in vitamin B12 biosynthesis (ko00750; LDA score = 3.9, *p* < 0.001), while formula-feeding was associated with enhanced starch and sucrose metabolism (ko00500; LDA score = 4.2, *p* < 0.001) and specific enrichment of iron complex outer membrane receptor protein K02014 (LDA score = 4.21, *p* < 0.001). Rural residence was linked to methane metabolism (ko00680; LDA score = 3.6, *p* = 0.005) and plant polysaccharide degradation, while cesarean delivery was associated with branched-chain amino acid transporter KO1999 (LDA score = 3.65, *p* < 0.001).

Integration of taxonomic and functional data revealed key genus-pathway associations: *Bifidobacterium* with folate biosynthesis, *Escherichia-Shigella* with branched-chain amino acid transport in cesarean-delivered infants, and *Prevotella* with methane metabolism in rural populations (Supplementary Table S5). Random forest modeling confirmed the clinical relevance of these shifts, identifying LPS biosynthesis as the top predictor of severe outcomes (AUC = 0.82) and butanoate metabolism as predictive of milder disease (AUC = 0.79).

### 3.8. Clinical and Inflammatory Correlations

The taxonomic–functional relationships revealed critical links between microbiome composition, metabolic activity, and host inflammation. *Faecalibacterium* and *Bacteroides* exhibit strong negative correlations with systemic inflammation markers (CRP: *r* = −0.42, *p* = 0.0027; WBC count: *r* = −0.37, *p* < 0.05), reinforcing their role in maintaining gut homeostasis and immune tolerance. In contrast, *Escherichia-Shigella* and *Klebsiella* were positively correlated with proinflammatory markers (CRP: *r* = 0.51, *p* < 0.001; WBC: *r* = 0.48, *p* = 0.0057). Cesarean delivery was associated with reduced representation of primary bile acid biosynthesis (ko00120, *padj* = 0.0083), which may disrupt bile acid-mediated antimicrobial defense and lipid metabolism. Additionally, urban infants presented a greater abundance of antibiotic resistance genes (ko01501, *padj* = 0.0053), likely due to greater exposure to antimicrobial agents in urban environments.

The integration of LOS data with microbiome profiles revealed significant associations between microbial composition and hospitalization outcomes. Infants with prolonged LOS (≥7 days) exhibited a specific dysbiotic signature, defined by a significant increase in Proteobacteria abundance (r = 0.48, *p* = 0.002) concurrent with a significant decrease in *Akkermansia* (r = −0.52, *p* < 0.001). Multivariate analysis confirmed that the combination of cesarean delivery and formula feeding remained an independent predictor of extended LOS after controlling for disease severity (β = 2.3, 95% CI: 1.3–3.8, t = 3.2, *p* = 0.006).

## 4. Discussion

The gut microbiome plays a pivotal role in modulating host immunity and pathogen resistance, particularly in pediatric populations vulnerable to RVGE [1,58]. This study revealed significant associations between demographic factors, microbial community structure, and clinical outcomes in infants hospitalized with acute rotavirus infection. By integrating taxonomic profiling with functional pathway analysis, this study demonstrates how early-life exposures, including feeding practices, delivery mode, and geographic residence, shape microbiome composition and metabolic potential, thereby influencing disease severity.

The study cohort revealed notable demographic differences between RVGE patients and healthy controls. Compared with controls, RVGE cases included an increased proportion of females, younger infants, and more urban residents. This agrees with local incidence [58], and contrasts with global epidemiological patterns, where rotavirus typically shows male and rural predominance [59], suggesting possible region-specific or sampling-related factors. Formula feeding was significantly more common among RVGE patients, whereas delivery mode did not differ significantly between groups [60]. These findings underscore the potential influence of early-life feeding practices on gut microbiota development and RVGE susceptibility.

RVGE infants exhibit pronounced abnormalities, including elevated white blood cell counts, anemia, and elevated C-reactive protein (CRP) [61], reflecting a state of systemic inflammation [62]. CRP levels showed the strongest association with microbial dysbiosis, serving as a proxy for gut barrier disruption [40]. Severe dehydration is exclusively observed in RVGE infants and is linked to osmotic diarrhea from malabsorbed carbohydrates [63]. These clinical parameters collectively underscore the systemic impact of RVGE, which extends beyond gastrointestinal symptoms [64].

### 4.1. Influence of Age and Sex on Microbiome and Severity

The infant gut microbiome demonstrated significant age-dependent variations. Younger infants presented distinct microbial profiles characterized by a greater abundance of pathobionts and lower levels of beneficial taxa, patterns indicative of an immature and vulnerable gut ecosystem [4]. These findings align with the window of highest vulnerability before adaptive immunity fully matures [6,65]. Older infants showed gradual microbial maturation consistent with dietary diversification [66], although they remained dysbiotic compared with healthy controls. The negative correlation between microbial diversity and inflammatory markers indicates that RVGE-associated inflammation may itself impede normal microbiome developmental trajectories [67]. These age-stratified patterns highlight the dynamic nature of the infant gut microbiome and identify a critical window for potential microbiome-targeted interventions [68].

Sex-specific differences in microbiota structure were also observed. Male infants presented more pronounced dysbiosis, with significant enrichment of inflammatory genera that correlated with elevated clinical markers of disease severity. This aligns with prior studies indicating sex-based immunological differences that influence microbial colonization and response to infection [69]. Compared with male infants, female infants presented increased abundances of protective taxa and decreased levels of inflammatory markers, highlighting a protective hormonal or immunological influence. These findings position sex as an underrecognized determinant of early microbial programming and disease susceptibility.

### 4.2. Impact of Geographic Residence and Local Epidemiology

Geographic residence has a potentially substantial effect on microbial structure. Rural infants displayed increased levels of certain opportunistic pathogens, possibly reflecting broader environmental exposures. In contrast, urban infants had higher abundances of genera linked to dietary diversity. These geographic disparities highlight the necessity for region-specific public health strategies designed to optimize early-life microbiota and mitigate infection risk [70,71].

### 4.3. The Critical Role of Feeding Practices

Feeding modality has one of the strongest influences on microbial structure. Formula feeding was associated with reduced microbial diversity and enrichment of pathobionts. The absence of human milk oligosaccharides (HMOs) in formula, which are crucial for promoting the growth of keystone genera such as *Bifidobacterium*, likely contributes to this dysbiotic state [8,72]. The anti-inflammatory effects of *Bifidobacterium*, which are mediated through acetate production and immune modulation, are critical for rotavirus containment [73]. Conversely, formula-fed infants presented upregulated microbial pathways for starch metabolism, reflecting an adaptive shift to complex carbohydrates [74]. Mixed-fed infants exhibited an intermediate state of dysbiosis, potentially indicating only partial protection from breastfeeding [75]. These findings reinforce the clinical importance of exclusive breastfeeding for promoting microbial resilience and reducing disease severity, indicating its candidate promotion as a key microbiome-based RVGE prevention strategy [76,77].

The enrichment of Gammaproteobacteria is a recognized dysbiosis signature in early life, particularly in formula-fed infants, where taxa such as Klebsiella and Escherichia/Shigella often expand [78,79,80]. In diarrheal settings, these genera commonly increase, and Proteobacteria expansion is broadly linked to inflammatory gut states [80,81].

Within this context, *Dickeya*, a Gammaproteobacterium within Pectobacteriaceae, is primarily known as a plant-associated pathogen; reports from human infant stool are uncommon, so any apparent enrichment should be interpreted cautiously and ideally validated with shotgun metagenomics or culture, to exclude environmental or reagent contamination [82,83].

In contrast, SCFA-associated lineages, including classic butyrate-producing bacteria, support epithelial integrity and temper mucosal inflammation; their predicted depletion is repeatedly observed in inflammatory bowel disease and correlates with impaired barrier function [84,85].

Taken together, these findings indicate a formula-feed-associated shift toward proinflammatory conditions. Gammaproteobacteria (*Klebsiella, Escherichia/Shigella*, and *Dickeya*) alongside depletion of beneficial SCFA producers may mark a gut ecosystem that is less capable of resolving viral injury, potentially predisposing preterm infants to more severe rotavirus disease and underscoring the role of diet-driven microbiota in neonatal infectious susceptibility [79,86].

Our analysis identified feeding mode as the most robust demographic differentiator between the RVGE and control cohorts. This strong association is mechanistically explained by the profound impact of formula feeding on the infant gut microbiome, which in turn exacerbates rotavirus pathogenesis. Formula-fed infants in our study exhibited a microbiome primed for severe disease, characterized by the depletion of beneficial, immunomodulatory genera such as *Akkermansia* and *Faecalibacterium*, and the enrichment of proinflammatory pathobionts like *Escherichia-Shigella* and *Streptococcus* [87]. This configuration likely facilitates more severe disease through a dual mechanism: first, by impairing gut barrier integrity due to a loss of mucin-degrading (*Akkermansia*) and butyrate-producing (*Faecalibacterium*) bacteria, and second, by creating a proinflammatory milieu that amplifies the tissue damage caused by rotavirus replication [84,85,87]. In contrast, breastfeeding fosters a microbiome enriched in *Bifidobacterium* (r = −0.42 with breastfeeding), which supports barrier function and immune regulation, thereby mitigating pathogenesis and resulting in the milder clinical outcomes we observed [8,72,73].

### 4.4. The Potential Impact of Delivery Mode and Synergistic Risk Factors

The high cesarean section rate was primarily driven by medical indications like prior cesarean delivery, yet multilevel analysis and provider interviews identified significant non-clinical contributors. These included systemic factors such as convenience incentives, insufficient supervision, and inadequate guideline implementation, alongside clinical management factors like incomplete partograph use [88,89]. The delivery mode further modulated the microbial composition. Cesarean delivery disrupts normal microbial succession, leading to the enrichment of opportunistic pathogens and the depletion of beneficial taxa acquired during vaginal birth [90]. Cesarean-born infants miss critical microbial inoculation from the maternal vaginal and fecal microbiota, which appears to predispose them to overgrowth of pathogens linked to severe nosocomial infections [91]. A synergistic association was observed when cesarean delivery was combined with formula feeding, resulting in increased dysbiosis, with the lowest levels of beneficial mucin degraders and the highest levels of pathobionts [87]. These results emphasize the need for strategies such as targeted probiotics to restore a healthy microbiome in cesarean-delivered infants [67].

### 4.5. Core Taxonomic and Functional Dysbiosis in RVGE

Rotavirus-infected infants presented significantly reduced microbial alpha diversity, which was strongly inversely correlated with both systemic inflammation and dehydration severity. This dysbiosis pattern aligns with established mechanisms linking microbial disruption to impaired viral clearance and prolonged duration of diarrhea [3,92].

Phylum-level analysis revealed a profoundly disrupted Firmicutes-to-Bacteroidetes (F/B), characterized by a marked depletion of Bacteroidetes and dominance of Firmicutes, with the severity of this imbalance tracking directly with clinical dehydration status [15,93]. The enrichment of Proteobacteria in severely dehydrated infants aligns with global studies linking this phylum to inflammatory gut dysbiosis [80,94,95]. Its association with inflammation supports its proposed role as a “pathobiont” that thrives in, and potentially exacerbates, disrupted gut environments [80]. Our machine learning results are consistent with emerging trends in microbiome diagnostics, where Proteobacteria consistently emerge as a top biomarker for gastrointestinal pathology [96], warranting further investigation into its potential as a therapeutic target [97].

The dysbiosis was multilayered, as evidenced by (1) the elevation of inflammation-associated genera that exacerbate symptoms through LPS production [63,98]; (2) the critical depletion of mucin-degrading taxa, impairing gut barrier function; and (3) the reduction in key anti-inflammatory butyrate producers and immunomodulatory taxa [36,99]. These collective microbial disruptions highlight the microbiome’s central immunomodulatory role in RVGE pathogenesis [87] and strongly advocate the development of microbiome restoration strategies as a compelling strategic direction [100,101,102].

### 4.6. Underlying Metabolic Shifts and Clinical Implications

Functional profiling revealed distinct metabolic alterations associated with RVGE. Phylum-level analysis revealed profound microbial dysbiosis in the RVGE gut microbiome, characterized by a marked dominance of Firmicutes and a significant bloom of Proteobacteria in cases of severe dehydration [63]. This shift in the taxonomic landscape was closely linked to functional metabolic deficiencies, notably a deficit in butyrate production. Concurrently, the depletion of beneficial taxa involved in vitamin synthesis and anti-inflammatory processes further underscores the disrupted metabolic equilibrium [103].

A core finding was the significant upregulation of nutrient scavenging pathways, such as ABC transporters, alongside a collective depression in protective metabolic functions. This includes impaired biosynthesis of short-chain fatty acids (SCFAs) and folate, coupled with an increase in proinflammatory pathways such as LPS biosynthesis and proteolysis [93,104,105]. These impairments collectively contribute to compromised nutrient absorption and a loss of immunoregulation. Furthermore, specific demographic exposures shaped the functional capacity of the microbiome. Formula feeding was associated with an increase in carbohydrate and protein metabolism, whereas cesarean delivery correlated with altered bile acid and xenobiotic degradation pathways, suggesting an adaptation to distinct early-life niches [106,107,108,109]. Conversely, breastfeeding enriched pathways essential for vitamin biosynthesis and SCFA production, and rural residence was linked to increased metabolic capabilities for fiber degradation [73,110].

The functional implications of these taxonomic and metabolic shifts are profound. The depletion of SCFA producers likely impairs gut barrier integrity and immune tolerance, whereas the enrichment of LPS-producing pathobionts may drive systemic inflammation [104]. Cesarean delivery-associated reductions in bile acid metabolism could further disrupt lipid absorption and microbial homeostasis [111], and the enrichment of xenobiotic degradation pathways in urban infants may reflect environmental pollutant exposure with potential long-term health consequences [112]. These findings demonstrate that RVGE pathophysiology is associated with a collective microbial metabolic shift away from homeostasis and toward inflammation. This underscores the potential for early-life interventions, such as breastfeeding or targeted microbiota restoration, to recalibrate these dysfunctional metabolic trajectories, offering novel therapeutic avenues for mitigating enteric infections [113,114].

### 4.7. Clinical Relevance and Therapeutic Implications

Our findings hold significant translational potential for the clinical management of pediatric rotavirus infection. The microbial signatures we identified could serve as valuable tools for risk stratification and the development of targeted interventions [20]. For instance, the consistent depletion of *Akkermansia*, *Faecalibacterium*, and *Bifidobacterium*, coupled with the enrichment of *Proteobacteria* and *Escherichia-Shigella*, provides a candidate biomarker profile for identifying infants at high risk of severe RVGE [20,115]. In a clinical setting, a rapid microbiome assessment at diagnosis could potentially flag these high-risk patients for more intensive monitoring and supportive care, optimizing resource allocation and potentially improving outcomes.

Furthermore, our data suggest concrete pathways for microbiome-based interventions to mitigate disease severity and promote recovery. The profound depletion of keystone taxa like *Akkermansia* and butyrate producers (*Faecalibacterium*, *Roseburia*) points to specific functional deficits—namely, impaired mucosal barrier integrity and reduced anti-inflammatory SCFA production—that could be therapeutically targeted [116].

Targeted Probiotics and Synbiotics: Our results strongly advocate for the development of next-generation probiotics and synbiotics tailored to the specific dysbiosis of RVGE and the patient’s demographic background. For example, probiotic formulations containing *Akkermansia muciniphila* or *Faecalibacterium prausnitzii* could be evaluated for their ability to restore gut barrier function and reduce inflammation [117,118]. Similarly, *Bifidobacterium* strains, which were strongly associated with breastfeeding and milder disease, could be administered to formula-fed or cesarean-delivered infants as a preventive or adjunctive therapy. These could be combined with prebiotics (e.g., human milk oligosaccharide analogs, fructooligosaccharides, or specific mucins) that selectively nourish these beneficial taxa, creating a synergistic synbiotic approach [119,120].

Dietary Modulations: The powerful protective association of breastfeeding underscores that diet is a primary lever for modulating the gut microbiome. For non-breastfed infants, our functional data, which show a loss of butanoate metabolism, suggest that nutritional interventions with prebiotic fibers that promote SCFA production (e.g., galactooligosaccharides, resistant starch) could be beneficial. In the convalescent phase, a dietary regimen designed to “re-boot” a healthy microbiome—rich in the fibers and nutrients that support *Bifidobacterium* and *Akkermansia*—could potentially shorten the duration of dysbiosis and accelerate full recovery [21,121].

### 4.8. Limitations

Several limitations of this study should be considered. The cross-sectional design captures a single time point during acute infection, which restricts our ability to infer causal relationships between the observed microbiome states and disease progression or to track microbial recovery longitudinally. While 16S rRNA sequencing provides robust community profiling, it lacks the strain-level resolution and functional precision offered by shotgun metagenomics, potentially omitting critical virulence factors or metabolic capabilities. It is important to note that the functional metabolic pathways (KOs) discussed herein are predictions based on 16S rRNA data via Tax4Fun and were not directly measured by metagenomic sequencing. Although stringent inclusion criteria controlled for major confounders such as recent antibiotic use, other unmeasured variables, such as detailed dietary history, household size, or specific environmental exposures, could influence microbiome composition. The study was conducted within a single geographic region, which may limit the generalizability of the findings to populations with different genetic backgrounds, diets, and healthcare practices. Furthermore, the reliance on parent-reported data for some demographic factors may introduce recall bias. Our analysis lacked viral load quantification or strain-level rotavirus characterization, which could influence disease severity. The potential impact of the hospital environment itself on the microbiome, particularly for infants delivered by cesarean section, was not assessed. Finally, while the use of multiple statistical and machine learning approaches strengthens biomarker identification, the predictive models require validation in independent, prospective cohorts to confirm their clinical utility.

## 5. Conclusions

This cross-sectional study indicates that early-life demographic factors are associated with distinct gut microbiome configurations during RVGE and that these configurations track clinical severity. We identified a signature of RVGE-associated dysbiosis characterized by a loss of microbial diversity, a disrupted Firmicutes–Bacteroidetes ratio, the depletion of beneficial commensals (*Akkermansia*, *Faecalibacterium*, and *Bifidobacterium*), and the enrichment of proinflammatory pathobionts (*Escherichia–Shigella*, and *Klebsiella*). Furthermore, integrated functional analysis revealed a collective shift in microbial metabolism away from protective functions such as butyrate synthesis and inflammatory pathways such as LPS biosynthesis. The convergence of multiple machine learning models on specific taxa and pathways as candidate biomarkers underscores the role of the microbiome as a key mediator of clinical outcomes. These findings provide a mechanistic basis for the observed disparities in RVGE severity and offer a compelling rationale for developing targeted, microbiota-based interventions. Promoting breastfeeding and vaginal delivery, alongside developing next-generation probiotics and prebiotics tailored to high-risk demographic groups, represents a promising strategic direction for reducing the global burden of pediatric rotavirus infection. Finally, our findings contribute to an accumulating body of evidence positioning the gut microbiome as a critical mediator of rotavirus outcomes, with implications for public health strategies in vulnerable populations.

## Figures and Tables

**Table 1:** Demographic and Clinical Characteristics of Rotavirus-Infected Infants (RVGE) vs. Healthy Controls.

Variable	Category	RVGE (*n* = 120)	Healthy (*n* = 45)	Effect Size (95% CI)	Test Statistic	*p*-Value
**Demographic Factors**						
Sex, n (%)	Male	45 (37.5%)	21 (46.7%)	OR = 0.68 (0.34–1.35)	χ^2^ = 1.12	0.29
	Female	75 (62.5%)	24 (53.3%)			
Age (months)	Mean ± SD	5.8 ± 3.2	6.9 ± 3.8	MD = −1.1 (−2.3–0.1)	t = −1.82	0.071
Residence, n (%)	Rural	48 (40.0%)	22 (48.9%)	OR = 0.70 (0.35–1.39)	χ^2^ = 1.06	0.30
	Urban	72 (60.0%)	23 (51.1%)			
Feeding Mode, n (%)	Formula	56 (46.7%)	9 (20.0%)	--	χ^2^ = 9.87	0.002
	Breast	58 (48.3%)	31 (68.9%)			
	Mixed	6 (5.0%)	5 (11.1%)			
Delivery Mode, n (%)	Cesarean	85 (70.8%)	34 (75.6%)	OR = 0.79 (0.36–1.75)	χ^2^ = 0.31	0.58
	Vaginal	35 (29.2%)	11 (24.4%)			
**Clinical and Laboratory Parameters**						
WBC (×10^3^/µL)	Mean ± SD	11.2 ± 3.1	6.1 ± 2.0	MD = 5.1 (4.2–6.0)	t = 11.2	<0.001
Hemoglobin (g/dL)	Mean ± SD	11.0 ± 1.3	12.3 ± 0.8	MD = −1.3 (−1.7–−0.9)	t = −6.87	<0.001
Platelets (×10^3^/µL)	Mean ± SD	510 ± 98	425 ± 50	MD = 85 (56–114)	t = 5.78	<0.001
CRP (mg/L)	Median [IQR]	1.6 [0.9–2.2]	0.5 [0.3–0.7]	--	U = 985.5	<0.001
Dehydration Status, n (%)	None	36 (30.0%)	45 (100%)	--	χ^2^ = 72.0	<0.001
	Moderate	76 (63.3%)	0 (0%)			
	Severe	8 (6.7%)	0 (0%)			

Abbreviations: CI, Confidence Interval; OR, Odds Ratio; MD, Mean Difference; SD, Standard Deviation; IQR, Interquartile Range; WBC, White Blood Cell count; CRP, C-reactive Protein.

## Data Availability

Raw sequencing data were deposited in the NCBI Sequence Read Archive (SRA) under BioProject PRJNA1251983. R scripts are available at https://gist.github.com/Mohammedramadan2012.

## Supplementary Materials

The following supporting information can be downloaded at: https://www.mdpi.com/article/10.3390/v17121542/s1, Table S1: Demographic and Clinical Characteristics of Rotavirus-Infected Infants (RVGE) vs. Healthy Controls. Table S2: Length of Stay Analysis by Demographic and Clinical Factors in RVGE Cohort. Table S3: Berger–Parker Index of Microbial Community Dominance Across Demographic and Clinical Variables. Table S4: Machine Learning Classifier Performance Metrics. Table S5: KEGG Orthologs (KOs) significantly associated with bacterial genera and patient demographics. Figure S1. Bacterial diversity analysis: Rarefaction curves. Figure S2. Machine Learning Analysis of Microbiome-Based Feeding Mode Classification. Figure S3. Receiver Operating Characteristic (ROC) curves showing the performance of the top discriminative bacterial genera in classifying infants by mode of delivery. Figure S4. Receiver Operating Characteristic (ROC) curves showing the performance of the top discriminative bacterial genera in classifying infants by feeding practices.
