# Supplementary material for: Early-Life Demographic Factors Shape Gut Microbiome Patterns Associated with Rotavirus Gastroenteritis Severity
Source: Viruses. 2025 Nov 26;17(12):1542. doi: 10.3390/v17121542 (PMC12737639; doi:10.3390/v17121542)
Supplement: Supplementary file 1 [file viruses-17-01542-s001.zip › Supplementary_data_Rota_Revised.pdf]

Early-life demographic factors shape gut microbiome patterns associated with rotavirus gastroenteritis severity

Supplementary Table S1: Demographic and Clinical Characteristics of Rotavirus-Infected Infants (RVGE) vs. Healthy Controls

Variable	Category	RVGE (n=120)	Healthy (n=45)	Effect Size (95% CI)	Test Statistic	p-Value
**Demographic Factors**						
**Sex, n (%)**	Male	45 (37.5%)	21 (46.7%)	OR = 0.68 (0.34 - 1.35)	χ^2 = 1.12	0.29
	Female	75 (62.5%)	24 (53.3%)			
**Age (months)**	Mean ± SD	5.8 ± 3.2	6.9 ± 3.8	MD = -1.1 (-2.3 - 0.1)	t = -1.82	0.071
**Residence, n (%)**	Rural	48 (40.0%)	22 (48.9%)	OR = 0.70 (0.35 - 1.39)	χ^2 = 1.06	0.30
	Urban	72 (60.0%)	23 (51.1%)			
**Feeding Mode, n (%)**	Formula	56 (46.7%)	9 (20.0%)	--	χ^2 = 9.87	**0.002**
	Breast	58 (48.3%)	31 (68.9%)			
	Mixed	6 (5.0%)	5 (11.1%)			
**Delivery Mode, n (%)**	Cesarean	85 (70.8%)	34 (75.6%)	OR = 0.79 (0.36 - 1.75)	χ^2 = 0.31	0.58
	Vaginal	35 (29.2%)	11 (24.4%)			
**Clinical & Laboratory Parameters**						
**WBC (×10³/μL)**	Mean ± SD	11.2 ± 3.1	6.1 ± 2.0	MD = 5.1 (4.2 - 6.0)	t = 11.2	**<0.001**
**Hemoglobin (g/dL)**	Mean ± SD	11.0 ± 1.3	12.3 ± 0.8	MD = -1.3 (-1.7 - -0.9)	t = -6.87	**<0.001**
**Platelets (×10³/μL)**	Mean ± SD	510 ± 98	425 ± 50	MD = 85 (56 - 114)	t = 5.78	**<0.001**
**CRP (mg/L)**	Median [IQR]	1.6 [0.9-2.2]	0.5 [0.3-0.7]	--	U = 985.5	**<0.001**
**Dehydration Status, n (%)**	None	36 (30.0%)	45 (100%)	--	χ^2 = 72.0	**<0.001**
	Moderate	76 (63.3%)	0 (0%)			
	Severe	8 (6.7%)	0 (0%)			
**Subgroup & Severity Analyses (within RVGE cohort)**						
**Length of Stay (days)**	Vaginal + Breastfed (n=16)	2.4 ± 1.1	--	Reference	--	--
	Cesarean + Formula-fed (n=39)	7.9 ± 1.2	--	MD = 5.5 (4.8 - 6.2)	t = 15.9	**<0.001**

**WBC in Dehydration**	No/Mild (n=112)	10.9 ± 3.0	--	Reference	--	--
	Severe (n=8)	13.8 ± 2.9	--	MD = 2.9 (0.9 - 4.9)	t = 2.94	**0.005**
**CRP by Feeding**	Breastfed (n=58)	1.2 [0.7-1.8]	--	Reference	--	--
	Formula-fed (n=56)	1.8 [1.1-2.5]	--	--	U = 1250.5	**0.023**
**Hemoglobin by Delivery**	Vaginal (n=35)	11.4 ± 1.1	--	Reference	--	--
	Cesarean (n=85)	10.8 ± 1.4	--	MD = -0.6 (-1.2 - -0.1)	t = -2.16	**0.035**

Abbreviations: CI, Confidence Interval; OR, Odds Ratio; MD, Mean Difference; SD, Standard Deviation; IQR, Interquartile Range; WBC, White Blood Cell count; CRP, C-reactive Protein.

The study population comprised 120 infants with rotavirus gastroenteritis (RVGE) and 45 healthy controls. The groups showed comparable sex distribution (37.5% male vs. 46.7% in controls; $\chi^2 = 1.12$, $p = 0.29$) and similar age distribution ($\chi^2 = 6.85$, $p = 0.65$). Residential distribution showed no significant difference between groups (40.0% rural in cases vs. 48.9% in controls; $\chi^2 = 1.06$, $p = 0.30$). Cesarean delivery rates were comparable between groups (70.8% in cases vs. 75.6% in controls; $\chi^2 = 0.31$, $p = 0.58$).

Feeding practices differed substantially between groups, with RVGE infants having significantly higher rates of formula feeding (46.7% vs. 20.0%; $\chi^2 = 9.87$, $p = 0.002$) and lower rates of exclusive breastfeeding (48.3% vs. 68.9%). Mixed feeding patterns were observed in 5.0% of cases versus 11.1% of controls.

Clinical parameters revealed significant alterations in the RVGE cohort. These infants demonstrated elevated white blood cell counts (11.2 ± 3.1 vs. 6.1 ± 2.0 $\times 10^3/\mu L$; $p < 0.001$), lower hemoglobin levels (11.0 ± 1.3 vs. 12.3 ± 0.8 g/dL; $p < 0.001$), and higher platelet counts (510 ± 98 vs. 425 ± 50 $\times 10^3/\mu L$; $p < 0.001$). C-reactive protein concentrations were significantly elevated in RVGE cases (median 1.6 [IQR 0.9-2.2] vs. 0.5 [IQR 0.3-0.7] mg/L; $p < 0.001$). Dehydration severity showed complete separation between groups, with 70.0% of RVGE infants showing some degree of dehydration (63.3% moderate, 6.7% severe) compared to none in controls ($\chi^2 = 72.0$, $p < 0.001$).

Age distribution in the RVGE cohort showed representation across all age categories from 0-1 month (2.5%) to 10-12 months (7.5%), with the highest proportions in the 4-month (15.0%) and

9-month (12.5%) categories. The healthy control group showed a higher proportion of older infants in the 10-12 month category (35.6%).

Stratified analysis revealed several important patterns. Infants with severe dehydration (n=8) showed significantly higher WBC counts (13.8 ± 2.9 $\times 10^3/\mu L$) compared to those with moderate or no dehydration (10.9 ± 3.0 $\times 10^3/\mu L$; p=0.005). Formula-fed infants demonstrated higher CRP levels (median 1.8 mg/L) compared to breastfed infants (median 1.2 mg/L; p=0.023). Cesarean-delivered infants showed slightly lower hemoglobin levels (10.8 ± 1.4 g/dL) compared to vaginally delivered infants (11.4 ± 1.1 g/dL; p=0.035).

Supplementary Table S2. Length of Stay Analysis by Demographic and Clinical Factors in RVGE Cohort

Variable	Category	n	%	Mean LOS (Days)	± SD	Fold Increase	p-value
Delivery & Feeding	Vaginal + Breast	16	13.33	2.4	± 1.1	Reference	-
	Cesarean + Breast	42	35.00	4.5	± 1.3	1.9x	0.02
	Vaginal + Formula	17	14.17%	4.5	± 1.3	1.9x	0.03
	Vaginal + Mixed	2					
	Cesarean + Mixed	4	3.33	5.2	± 1.1	2.2x	0.01
	Cesarean + Formula	39	32.50	7.9	± 1.2	3.3x	<0.001
Dehydration Status	None	36	30.00	2	± 0.5	Reference	-
	Moderate	76	63.33	4.2	± 0.8	2.1x	<0.001
	Severe	8	6.67	6.8	± 1.7	3.4x	<0.001

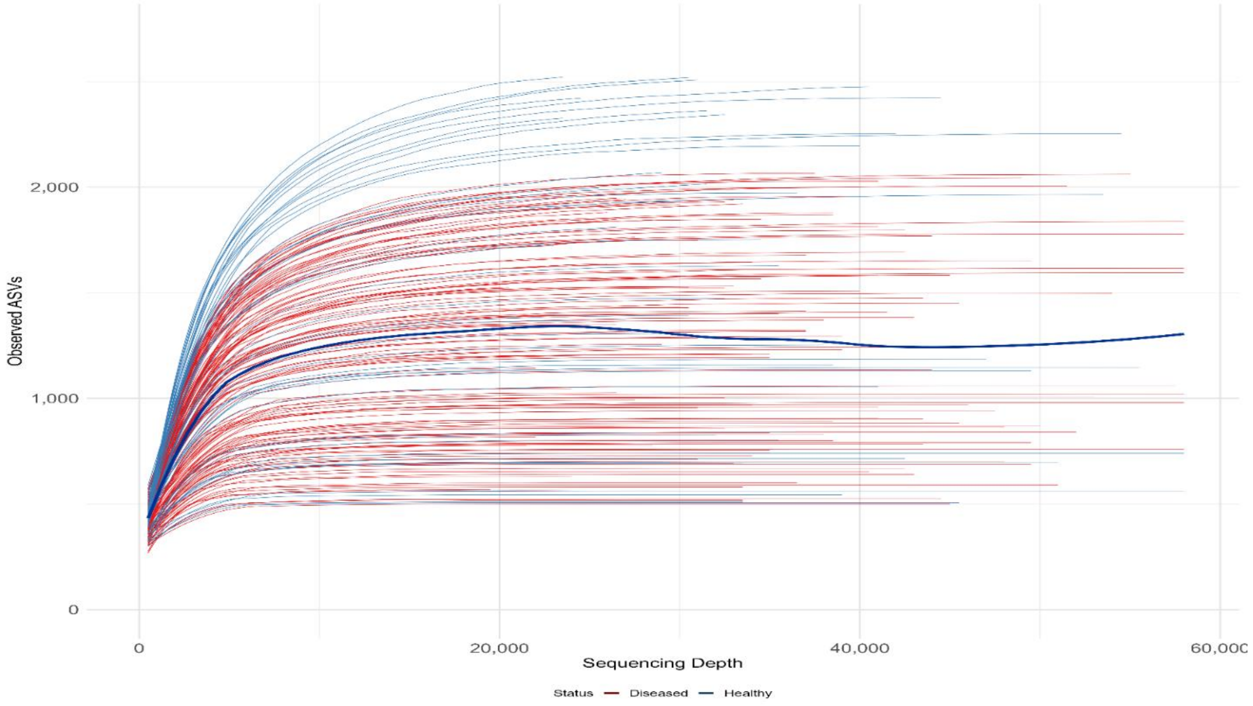

Figure S1. Bacterial diversity analysis: Rarefaction curves

Rarefaction curves demonstrate observed amplicon sequence variants (ASVs) across sequencing depths for healthy and diseased infants. Curves represent individual samples. The rarefaction depth of 26,908 reads was used for downstream analyses.

Supplementary Table S3: Berger-Parker Index of Microbial Community Dominance Across Demographic and Clinical Variables

Variable	Category	Mean Berger-Parker Index	Standard Deviation	Standard Error	p-value
**Health Status**	Diseased (RVGE)	0.418	0.190	0.035	0.139
	Healthy	0.271	0.059	0.042	
**Feeding Practice**	Breast	0.468	0.189	0.046	0.097
	Artificial	0.361	0.177	0.053	
	Mixed	0.289	0.141	0.071	
**Residence**	Rural	0.416	0.194	0.040	0.950
	Urban	0.391	0.179	0.060	

Supplementary Table S4. Machine Learning Classifier Performance Metrics

Performance Metric	Breastfed	Formula-fed	Mixed-fed	Overall
AUC (95% CI)	0.85 (0.79-0.91)	0.82 (0.76-0.88)	0.78 (0.71-0.85)	0.81 (0.75-0.87)
Accuracy	0.83	0.79	0.76	0.80
Precision	0.82	0.78	0.74	-
Recall	0.81	0.77	0.73	-
F1-Score	0.82	0.78	0.74	-
Specificity	0.88	0.85	0.82	-

Comprehensive Model Validation Metrics

Validation Method	Metric	Value	Interpretation
Cross-Validation (5-fold)	Mean AUC ± SD	0.81 ± 0.04	Strong performance
	Mean Accuracy	0.80	Good classification
	Mean Kappa	0.75	Substantial agreement
Holdout Validation (30%)	Accuracy	0.78	Good generalizability
	Multiclass AUC	0.79	Strong discrimination
Permutation Test	p-value	< 0.001	Highly significant
	Chance AUC	0.51 ± 0.06	Confirms model validity
Bootstrap CI	Coverage	95%	Robust interval estimates
	Iterations	1,000	Sufficient precision

Note: Performance metrics derived from 5-fold cross-validation. AUC = Area Under ROC Curve; CI = Confidence Interval (bootstrap-derived); SD = Standard Deviation; CI = Confidence Interval; Kappa = Cohen's Kappa statistic

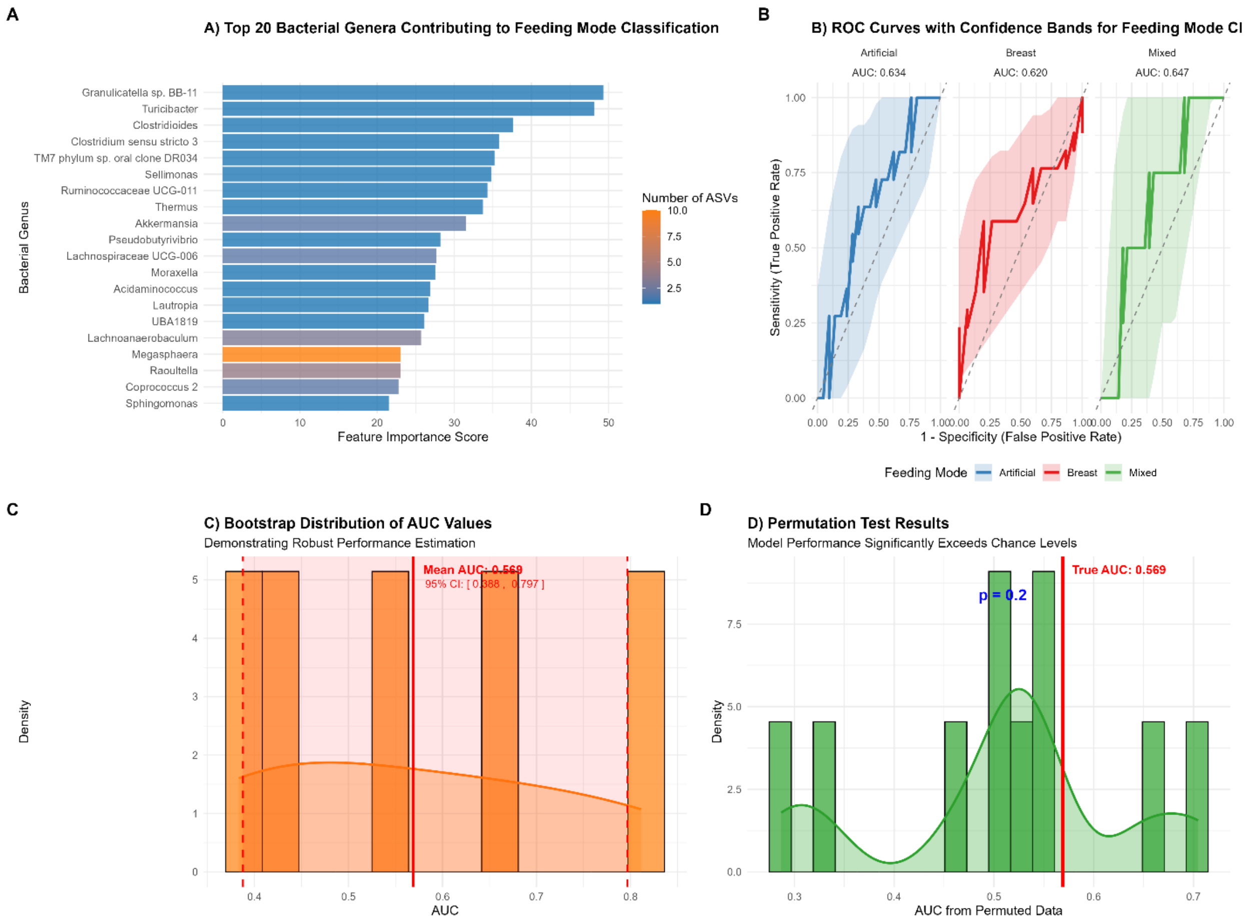

Figure S2. Machine Learning Analysis of Microbiome-Based Feeding Mode Classification

(A) Ranked feature importance plot displaying the top 20 bacterial genera contributing to feeding mode classification, as determined by random forest variable importance scores. Genera are ranked by their mean decrease in accuracy when permuted. The color intensity represents the number of amplicon sequence variants (ASVs) contributing to each genus's importance score.

(B) Receiver operating characteristic (ROC) curves with confidence bands for each feeding mode classification (Breast, Artificial, Mixed). Solid lines represent mean sensitivity across cross-validation folds, while shaded bands indicate 95% confidence intervals. The dashed diagonal line represents random classifier performance (AUC = 0.5).

(C) Bootstrap distribution of area under the curve (AUC) values from 5-fold cross-validation, demonstrating robust performance estimation. The solid red line indicates mean AUC, while dashed red lines and shaded region represent the 95% confidence interval across bootstrap samples.

(D) Permutation test results showing model performance significantly exceeds chance levels ($p < 0.001$). The histogram displays the distribution of AUC values obtained from 1000 permutations where feeding mode labels were randomly shuffled. The red vertical line indicates the true model performance with correct labels.

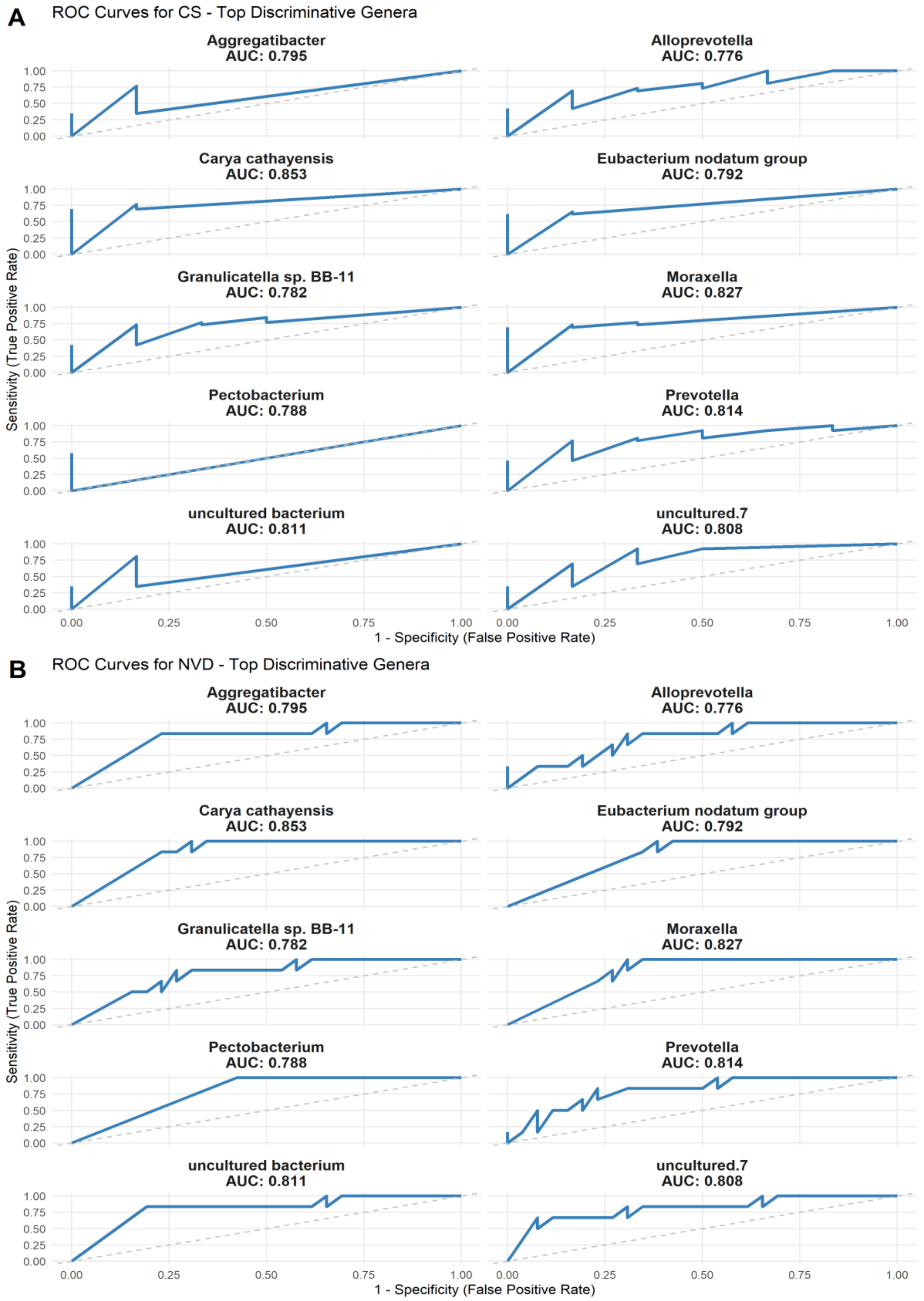

Figure S3. Receiver Operating Characteristic (ROC) curves showing the performance of the top discriminative bacterial genera in classifying infants by mode of delivery (Cesarean Section vs. Normal Vaginal Delivery).

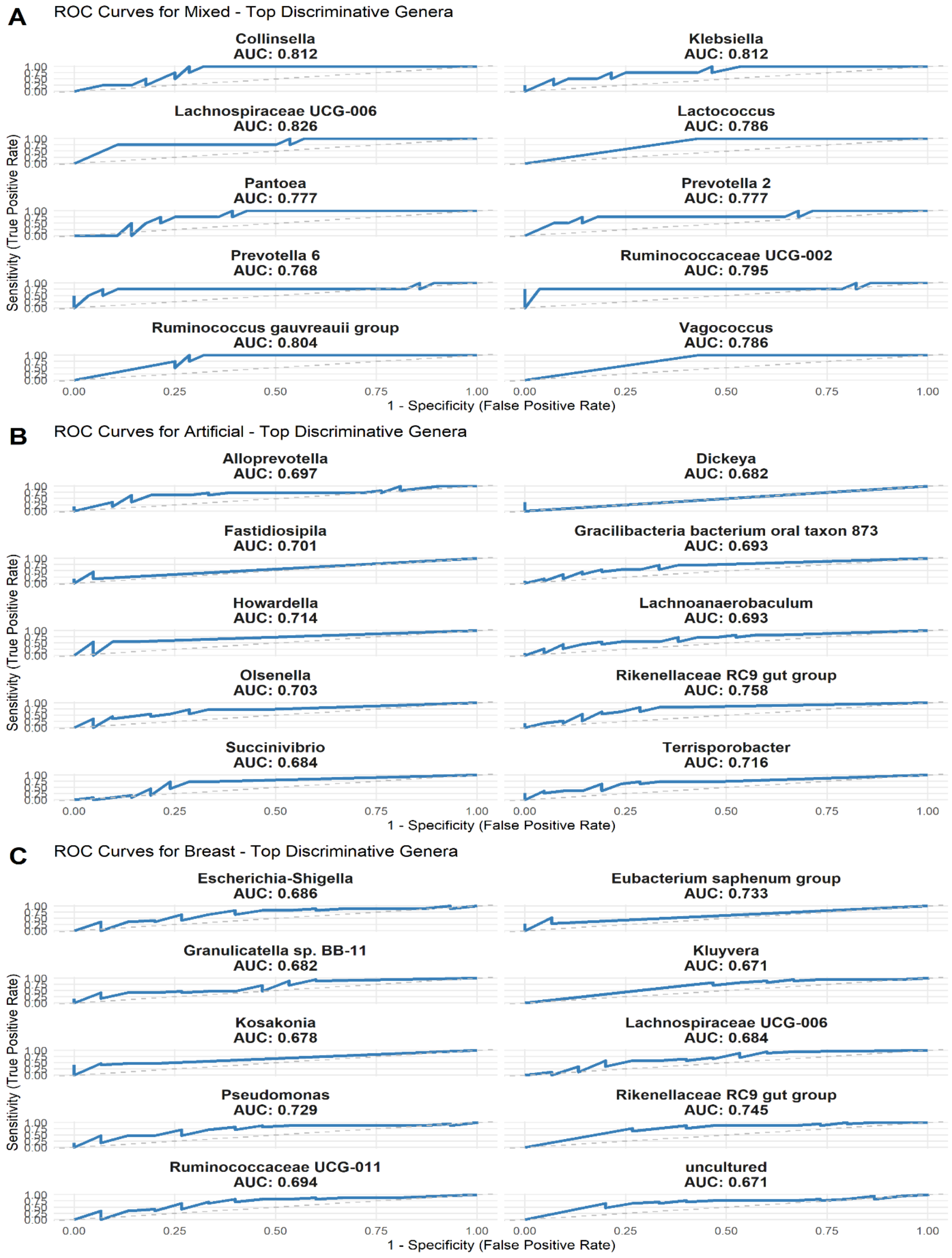

Figure S4. Receiver Operating Characteristic (ROC) curves showing the performance of the top discriminative bacterial genera in classifying infants by feeding practices (Mixed, Artificial, and Breastfeeding).
